# Association of work-related psychosocial factors and day-to-day home blood pressure variation: the Finn-Home study

**DOI:** 10.1097/HJH.0000000000003619

**Published:** 2023-11-15

**Authors:** Saana Karelius, Jaana Pentti, Eeva Juhanoja, Antti Jula, Seppo Koskinen, Teemu J. Niiranen, Sari Stenholm

**Affiliations:** a Department of Internal Medicine, University of Turku; b Division of Medicine, Turku University Hospital; c Department of Public Health, University of Turku and Turku University Hospital; d Centre for Population Health Research, University of Turku and Turku University Hospital, Turku; e Clinicum, Faculty of Medicine, University of Helsinki, Helsinki, Finland; f Oncology Ward, Operational Division of Surgery and Cancer Diseases, Turku University Hospital, Turku; g Department of Public Health Solutions, Finnish Institute for Health and Welfare, Helsinki; h Research Services, Turku University Hospital and University of Turku, Turku, Finland

**Keywords:** home blood pressure, job control, job demands, workers

## Abstract

**Objectives:**

Stress, and particularly job strain, has been found to associate with ambulatory blood pressure (BP). Moreover, BP is known to vary between days. One potential over-looked factor underlying this day-to-day BP variation could be work-related psychosocial factors. Thus, we aimed to study the association between job strain, job demands, job control and day-to-day BP variation.

**Methods:**

The home BP of 754 regularly working participants (mean age 50.9 ± 4.8, women 51%) of the Finn-Home Study was measured twice in the morning and twice in the evening over seven days. Average SBP and DBP were calculated for each day. Work-related psychosocial factors were measured with survey. Multivariable-adjusted generalized linear models were used for statistical analysis.

**Results:**

We found a greater SBP/DBP decrease between weekdays and weekend among participants with high job strain (-1.8 [95% confidence interval, 95% CI, -2.7 to -0.8]/-1.7 [95% CI, -2.3 to -1.1] mmHg) compared to participants with low job strain (-0.7 [95% CI, -1.1 to -0.2]/-0.7 [95% CI, -1.0 to -0.4] mmHg). The participants with high job demands showed a higher BP decrease between weekdays and weekend (-1.4 [95% CI, -2.0 to -0.8]/-1.3 [95% CI, -1.6 to -0.9] mmHg) than the participants with low job demands (-0.5 [95% CI, -1.1 to 0.0]/-0.6 [95% CI, -1.0 to -0.3] mmHg). We did not find BP differences regarding job control.

**Conclusion:**

High job strain and high job demands were associated with a greater BP reduction from weekdays to the weekend. Work-related psychosocial factors should be considered when assessing day-to-day BP variation.

## INTRODUCTION

Job strain is associated with an increased ambulatory blood pressure (BP), particularly in men [1]. This may be partially dependent on the day of the week as ambulatory BP is known to vary during the week from working days to days off [2–6]. According to most of these studies, BP is higher during working days than during days off. Moreover, job strain is also associated with increased BP during leisure time and nighttime within a workday [7–9].

Despite the research on the associations between the day of the week and BP, only a few studies have examined whether work-related psychosocial factors, and especially job strain, associate with ambulatory BP differences between work days and days off work [4,9]. A Dutch study of 109 male white-collar workers demonstrated that high imbalance (a combination of high effort and low reward at work), but not overcommitment (an exhaustive work-related coping style indexing the incapability to unwind), was associated with higher SBP values not only during workdays but also during leisure days [9]. Another study from the Netherlands analyzing 159 female nurses reported that ambulatory BP was higher during workdays than during leisure days, but they did not find an association between job strain and BP on workdays or leisure days [4].

Home BP is currently a widely used BP measurement method that is a stronger predictor of cardiovascular events than office BP [10]. In addition, home BP measurement is currently recommended for long-term monitoring of treated hypertension [11] and is a more feasible method than office or ambulatory BP for assessing day-to-day BP variation. However, the association between work-related psychosocial factors and home BP remains largely unknown. We therefore studied the association between work-related psychosocial factors (job strain, job demands and job control) and day-to-day home BP variation among a large sample of employees in a wide range of occupations.

## MATERIALS AND METHODS

### Study participants

The study sample was drawn from the Health 2000 Study participants. The Health 2000 Study was conducted by the Public Health Institute of Finland in years 2000--2001. The country-wide population-based study sample (*n* = 8028) consisted of randomly selected individuals aged 30–99 years residing in Finland.

Details of the Health 2000 Study has been described earlier [12]. In total, 6986 individuals participated in a home interview. At the end of the home interview, 2108 individuals received a home BP monitor based on random availability of the monitors (Finn-Home study). The participants were instructed to measure their BP at home during seven consecutive days before the health examination that took place approximately three weeks later. Both the BP monitor and the BP measurement results were returned at the health examination. A more precise description of the Finn-Home study protocol has been previously reported elsewhere [13].

We excluded participants who had not measured their BP at least minimum of two times per day, on at least 2 weekdays (Monday, Tuesday, Wednesday, Thursday, Friday) and during both weekend days (Saturday and Sunday) (*n* = 63). Moreover, we excluded individuals in the following order: participants without full-time or part-time employment (*n* = 944), participants without a regular daytime job (*n* = 330) and participants who did not answer at least two job demands questions and four job control questions (*n* = 17). As a result, the data comprised 754 participants. The participants, who had sufficient number of BP measurements in mornings or in evenings separately (745 participants for morning measurements and 719 participants for evening measurements) and fulfilled the terms of previously described selection process otherwise, were included in the analysis that investigated the morning and evening BP values separately.

The Health 2000 Study was approved first in 1999 by the National Public Health Institute's Ethical Committee and then in 2000 by the Ethical Committee for Research in Epidemiology and Public Health at the Hospital District of Helsinki and Uusimaa [12]. All participants gave signed informed consent [12].

### Blood pressure measurements

Home BP was self-measured from the nondominant arm on seven consecutive days with an OMRON M4 monitor (Omron Matsusaka Co, Japan) after a 10-min rest [14] according to previous studies and current guidelines [11,15]. The participant rested for at least 5 min before the measurement with the cuff around the upper arm. Two different cuff sizes were used depending on the participants’ upper arm circumference. The participants were advised to measure BP twice in the morning and twice in the evening before taking their medication. Heavy exercise, smoking, drinking (except water) and eating were forbidden during the hour before the measurement. The participants marked the home BP measurement results on separate forms. For the main analyses, we used the mean value of all available measurements during a single day as the BP of a day. In addition, additional analyses were conducted by using mean value of morning measurements and evening measurements, respectively.

To compare the current study population to the larger part of the Health 2000 Study participants, information on the office BP measurements conducted at the health examination was used. The office BP in the health examination was taken by a standard mercury manometer (Mercuro 300; Speidel & Keller, Jungingen, Germany) preferably from the right arm and only exceptionally from the left arm with a convenient cuff. The participant was advised to avoid eating for four hours and to avoid physical exertion before the investigation and to sit silently in the measurement room for five minutes before the measurement. The mean of two measurements taken with two minutes break was used.

The use of antihypertensive medication was self-reported including diuretics (ATC codes C03, C03C, C03E), beta blockers (ATC codes C07A, C07B, C07F), calcium channel blockers (ATC codes C08) and angiotensin-converting enzyme (ACE) inhibitors and angiotensin II receptor blockers (ATC codes C09A, C09B, C09C, C09D).

### Work-related psychosocial factors

Job strain was measured with a modified version of the Job Content Questionnaire [16]. The following job demands items were used: ‘My job requires working very fast’; ‘My job requires working very hard’; ‘I am not asked to do an excessive amount of work’; ‘I have enough time to get my job done’; and ‘My job includes conflicting demands’. The following job control items were used: ‘My job allows me to make a lot of decisions on my own’; ‘My job requires me to be creative’; ‘My job requires that I learn new things’; ‘My job involves a lot of repetitive work’; ‘I have a lot of control over what happens at my job’; ‘My job requires a high level of skill’; ‘I get to do a variety of different things on my job’; ‘I have an opportunity to develop my own special abilities’; and ‘On my job, I am given a lot of freedom to decide how I do my work’. All items had a 5-point response scale, ranging from ‘I completely agree’ to ‘I completely disagree’. The participants were divided into high vs. low demands and high vs. low control based on median scores in the entire Health 2000 Study sample (3.20 for demands and 3.78 for control). The presence of high job strain was defined as having high demands (above the median) and low control (below the median). All other participants were included in the low job strain group.

### Covariates

Educational level was self-reported and classified as low, intermediate or high. Occupational status was classified as nonmanual (soldiers, directors and superior civil servants, specialists, experts, office and client service workers, service employees, sales and care workers), manual (farmers, foresters, construction, repair and craft workers and process and transport workers), and other workers. BMI was calculated from the measured weight and height. Information on physical activity, alcohol use and smoking was collected in a questionnaire. Self-reported leisure-time physical activity was classified into three categories based on the volume of activity: low, medium, and high [17]. Alcohol use was categorized as risk-use or not (≥288 g per week for men, ≥192 g per week for women) [18]. Smoking status was classified as current smoker (daily or less often) and nonsmoker (never and former).

### Statistical analyses

Characteristics of the participants are shown as mean values and standard deviation for continuous variables and frequencies for categorical variables. To compare characteristics of the participants by work-related psychosocial factors, we used *t*-test procedure for continuous variables and chi-squared test for categorical variables.

To assess the mean level of SBP and DBP values by day of the week across job strain, job demands and job control groups, we used a generalized linear model adjusted for age, sex and education.

For the main analyses we used linear regression analyses with generalized estimation equations (genmod procedure in SAS) to calculate the mean systolic and diastolic BP during weekdays and weekend days (time according to job strain, job demands and job control groups). To examine whether the difference in systolic and diastolic BP between weekdays and weekend was different among those with work-related psychosocial risk factor and those without the risk factor, we included an interaction term time∗work-related psychosocial factor in the model. The main analyses were first adjusted for age, sex, educational level and occupational status and then also for BMI, physical activity, smoking and alcohol use. We tested whether the results of the main analyses differed by sex by adding an interaction term time∗work-related psychosocial factor∗sex in the model. As a result, the interaction *P* values were always more than 0.05 demonstrating that there were no significant differences between women and men. To further explore the changes in BP values between weekdays and weekend days, the main analyses were performed separately for the morning and evening BP values. These analyses were adjusted for age, sex, educational level, occupational status, BMI, physical activity, smoking and alcohol use. Finally, to explore the differences in BP changes between participants with full-time and part-time employment, the sensitivity analysis among the participants with full-time employment was performed.

The statistical analyses were performed with SAS software, version 9.4 (SAS Institute Inc., Cary, North Carolina, USA).

## RESULTS

The characteristics of the study participants are presented in Table 1. Half of the participants (51%) were women and the mean age was 50.9 ± 4.8 years. Overall, 44% of the participants had high job demands, 50% of the participants had low job control and, eventually, 22% of the participants scored high job strain. In Supplementary Table 1, we present main characteristics of the participants by work-related psychosocial factors. High job strain and low job control were more common in women than in men, as well as among those with low education and manual occupation. No difference in antihypertensive medication or hypertension were observed across job strain, job demands and job control groups (Supplementary Table 1).

**TABLE 1:** Characteristics of the study participants

Characteristic	Participants (*n* = 754)
Women (*n*, %)	381 (51)
Age (mean, SD)	50.9 ± 4.8
Low education (*n*, %) Manual occupation (*n*, %)	202 (27) 146 (19)
High job demands (*n*, %)	329 (44)
Low job control (*n*, %)	374 (50)
High job strain (*n*, %)	167 (22)
BMI (kg/m^2^) (mean, SD)	26.9 ± 4.3
Low physical activity (*n*, %)	180 (24)
Excessive alcohol consumption (*n*, %)	64 (9)
Current smoker (*n*, %)	193 (26)
SBP, women (weekday, mmHg) SBP, men (weekday, mmHg) DBP, women (weekday, mmHg) DBP, men (weekday, mmHg) SBP, women (weekend, mmHg) SBP, men (weekend, mmHg) DBP, women (weekend, mmHg) DBP, men (weekend, mmHg)	120.3 (17.2) 130.0 (15.4) 77.5 (9.1) 82.5 (9.0) 119.2 (17.4) 129.3 (15.6) 76.6 (9.3) 81.5 (9.0)
Antihypertensive medication (n, %)	142 (21)
Hypertension (*n*, %)	245 (32)

Data are presented as number of participants (percentage) for class variables or as mean (SD) for continuous variables. BP, blood pressure; SD, standard deviation.

The characteristics of the participants who took part only in the health examination were generally similar to the characteristics of the participants in the current study sample (Supplementary Table 2).

**FIGURE 1:**
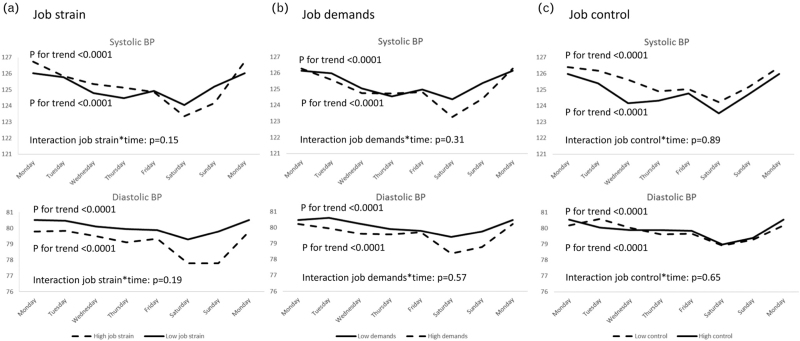
Day-to-day variability of blood pressure by (a) job strain, (b) job demands and (c) job control. Means are adjusted for age, sex and educational level. Trend tested for the BPs from Monday to Saturday. BP, Blood pressure.

Figure 1 visualizes the day-to-day SBP and DBP variability in groups by job strain, job demands and job control. SBP and DBP demonstrated a decreasing trend from Monday to Saturday in all subgroups (*P* for trend <0.0001), but there was no difference between groups. SBP and DBP increased from Saturday to Sunday, except for DBP in participants with high job strain as well as from Sunday to Monday.

Table 2 displays the multivariate-adjusted mean weekday and weekend BP by job strain, job demands and job control. There was no difference in SBP or DBP between participants with and without work-related psychosocial risk factors (high job strain, high job demands or low job control) either on weekdays or on weekend days. However, participants with high job strain demonstrated a significantly greater decrease in BP from weekdays to the weekend [SBP -1.8 mmHg, 95% confidence interval (95% CI) -2.7 to -0.8; DBP -1.7 mmHg, 95% CI -2.3 to -1.1], compared to the participants with low job strain (SBP -0.7 mmHg, 95% CI -1.1 to -0.2; DBP -0.7 mmHg, 95% CI -1.0 to -0.4]. Additionally, BP decreased more from weekdays to the weekend in participants with high job demands (SBP -1.4 mmHg, 95% CI -2.0 to -0.8; DBP -1.3 mmHg, 95% CI -1.6 to -0.9) than in participants with low job demands (SBP -0.5 mmHg, 95% CI -1.1 to 0.0; DBP -0.6 mmHg, 95% CI -1.0 to -0.3). In contrast, no difference in SBP or DBP was observed between weekdays and weekend among individuals with high and low job control. Supplementary Table 3 shows the corresponding BP values while adjusting for age, sex, educational level and occupational status. These results were similar to the main results (Table 2).

**TABLE 2:** Mean level and change between weekdays and weekend blood pressure by work-related psychosocial factors

BP	Work-related psychosocial factor	Weekday BP mean (95% CI)	Weekend BP mean (95% CI)	BP change between weekdays and weekend mean (95% CI)	*P* for interaction time∗work-related psychosocial factor
	Job strain				
Systolic	Low	127.8 (125.6–130.0)	127.2 (125.0–129.4)	-0.7 (-1.1 to -0.2)	0.039
	High	129.3 (126.3–132.4)	127.6 (124.6–130.6)	-1.8 (-2.7 to -0.8)	
Diastolic	Low	81.5 (80.2–82.8)	80.8 (79.5–82.1)	-0.7 (-1.0 to -0.4)	0.0028
	High	81.6 (79.9–83.3)	79.9 (78.3–81.6)	-1.7 (-2.3 to -1.1)	
	Job demands				
Systolic	Low	128.2 (125.9–130.4)	127.7 (125.4–129.9)	-0.5 (-1.1 to 0.0)	0.035
	High	128.2 (125.6–130.8)	126.8 (124.2–129.3)	-1.4 (-2.0 to -0.8)	
Diastolic	Low	81.6 (80.3–82.9)	81.0 (79.7–82.3)	-0.6 (-1.0 to -0.3)	0.020
	High	81.4 (79.9–82.8)	80.1 (78.7–81.5)	-1.3 (-1.6 to -0.9)	
	Job control				
Systolic	Low	128.9 (126.5–131.3)	128.0 (125.6–130.4)	-0.9 (-1.5 to -0.3)	0.92
	High	127.1 (124.6–129.5)	126.2 (123.8–128.6)	-0.9 (-1.5 to -0.3)	
Diastolic	Low	81.8 (80.4–83.2)	80.9 (79.5–82.3)	-0.9 (-1.3 to -0.5)	0.91
	High	81.1 (79.7–82.5)	80.1 (78.7–81.5)	-0.9 (-1.3 to -0.6)	

Means are adjusted for age, sex, educational level, occupational status, BMI, physical activity, smoking and alcohol use. BP, blood pressure; CI, confidence interval.

The further analyses comparing the weekday and weekend BP values separately for morning and evening BP values showed that the main results were mostly driven by morning, and not evening, BP values. These results adjusted for age, sex, educational level, occupational status, BMI, physical activity, smoking and alcohol use are summarized in Table 3.

**TABLE 3:** Mean level and change between weekdays and weekend blood pressure by work-related psychosocial factor separately for morning and evening values

BP	Work-related psychosocial factor	Weekday BP mean (95% CI)	Weekend BP mean (95% CI)	BP change between weekdays and weekend mean (95% CI)	*P* for interaction time∗work-related psychosocial factor
Morning					
	Job strain				
Systolic	Low	125.8 (123.4–128.2)	125.3 (122.9–127.7)	-0.5 (-1.1 to 0.1)	0.032
	High	127.5 (124.2–130.9)	125.5 (122.3–128.7)	-2.0 (-3.3 to -0.8)	
Diastolic	Low	81.2 (79.8–82.6)	80.6 (79.2–82.0)	-0.6 (-1.0 to -0.3)	0.0046
	High	81.5 (79.7–83.2)	79.6 (77.9–81.4)	-1.8 (-2.6 to -1.1)	
	Job demands				
Systolic	Low	126.1 (123.7–128.6)	125.9 (123.5–128.4)	-0.2 (-0.9 to 0.5)	0.010
	High	126.3 (123.5–129.1)	124.7 (121.9–127.4)	-1.6 (-2.4 to -0.8)	
Diastolic	Low	81.4 (79.9–82.8)	80.7 (79.3–82.2)	-0.7 (-1.1 to -0.2)	0.13
	High	81.1 (79.5–82.6)	79.9 (78.4–81.4)	-1.2 (-1.7 to -0.7)	
	Job control				
Systolic	Low	127.0 (124.4–129.6)	126.0 (123.4–128.6)	-1.0 (-1.7 to -0.2)	0.70
	High	125.1 (122.5–127.8)	124.4 (121.8– 127.0)	-0.7 (-1.5 to -0.0)	
Diastolic	Low	81.6 (80.1–83.1)	80.5 (79.0–82.0)	-1.1 (-1.6 to -0.6)	0.25
	High	80.8 (79.3–82.3)	80.1 (78.6–81.6)	-0.7 (-1.1 to -0.3)	
					
Evening					
	Job strain				
Systolic	Low	128.8 (126.4–131.1)	127.9 (125.5–130.2)	-0.9 (-1.5 to -0.3)	0.56
	High	130.0 (127.0–133.1)	128.8 (125.7–131.9)	-1.2 (-2.3 to -0.1)	
Diastolic	Low	81.1 (79.7–82.4)	80.3 (78.9–81.6)	-0.8 (-1.2 to -0.4)	0.14
	High	81.1 (79.3–82.8)	79.7 (78.0–81.4)	-1.4 (-2.0 to -0.7)	
	Job demands				
Systolic	Low	129.2 (126.8–131.6)	128.3 (125.9–130.7)	-0.9 (-1.6 to -0.2)	0.75
	High	129.0 (126.3–131.6)	127.9 (125.3–130.6)	-1.0 (-1.8 to -0.3)	
Diastolic	Low	81.2 (79.8–82.6)	80.5 (79.0–81.9)	-0.7 (-1.2 to -0.3)	0.15
	High	80.9 (79.4–82.4)	79.7 (78.2–81.2)	-1.2 (-1.6 to -0.7)	
	Job control				
Systolic	Low	129.9 (127.4–132.4)	129.0 (126.5–131.6)	-0.8 (-1.5 to -0.1)	0.65
	High	127.9 (125.4–130.5)	126.9 (124.3–129.5)	-1.1 (-1.8 to -0.3)	
Diastolic	Low	81.3 (79.8–82.8)	80.6 (79.1–82.1)	-0.7 (-1.1 to -0.2)	0.15
	High	80.6 (79.1–82.1)	79.5 (78.0–81.0)	-1.2 (-1.6 to -0.7)	

Means are adjusted for age, sex, educational level, occupational status, BMI, physical activity, smoking and alcohol use. BP, blood pressure; CI, confidence interval.

The results of the sensitivity analysis including only the participants with full-time employment (*n* = 707) are shown in Supplementary Table 4. These results replicated the results of the main analysis, indicating that the full-time workers did not differ from part-time workers regarding BP changes between weekdays and weekend in groups by work-related psychosocial factor.

## DISCUSSION

In this large population-based sample, we studied the association of work-related psychosocial factors with BP changes between weekdays and weekend. Our results demonstrate that individuals with high job strain or high job demands show a greater BP decrease in SBP and DBP between weekdays and weekend than participants with low job strain and low job demands. We observed no differences in BP changes from weekdays to weekend between groups by job control status.

The previous studies in this domain [2–5] offer some information on BP variation between work days and days off, but the study samples of these studies have been quite young (mean age range 27.2–37.8) and limited in size (30–159 participants). Moreover, the previous studies have mainly studied healthcare professionals. The results of the three previous studies that used ambulatory BP measurements indicate that higher BP values are observed during working days than on the days off [3–5]. Only one previous study did not find statistically significant differences in ambulatory BPs between work days and leisure days [2]. Additionally, the employed individuals have had higher BP values at the beginning of the week than during the weekend [6]. Moreover, in a few studies, job strain has associated with increased BP levels also in workday's leisure time and nighttime [7–9].

However, to the best of our knowledge, there are no previous studies that have analyzed the relation of work-related psychosocial factors and BP over a seven-day follow-up over a work-week. In a single previous study, the participants underwent ambulatory BP on one workday and on one day of leisure and filled in the Karasek job content questionnaire [4]. In that study, job strain was not associated with the ambulatory BP whereas high job demands were associated with a higher SBP on a work-day and not on a leisure day. Moreover, high job demands were associated with higher DBP at work but only when decision latitude (combination of skill discretion and decision authority) was also high. These results differ from the results of the current study, as we observed that both SBP and DBP seemed to decrease more from weekdays to the weekend in participants with high job strain and high job demands compared to their counterparts. This difference could partly attribute to the differences in study methodologies: the participants of the previous study were female nurses whose mean age was 36 years while our study sample included women and men from wide range of occupations with a mean age of 51 years. Moreover, the previous study measured the BP during a third consecutive day of morning shifts and during a second consecutive leisure day. In contrast, we compared the average home BP of five weekdays and two weekend days to each other.

Another previous Dutch study suggested that high imbalance but not overcommitment, was associated with higher SBP values not only during workdays but also during leisure days, whereas work-related psychosocial factors were not related to DBP [9]. Considering that the mean age of the Dutch study sample (47.2 ± 5.3 years) was close to the mean age of the current study sample, that individuals from white-collar occupations were included, and that the BP measurement methods resembled the methods of the current study (ambulatory BP measurement was performed on two work-days and on one leisure day), the somewhat differing results do not seem to stem from methodological factors.

One of the main findings of this study was, that the differences in home-measured BP between the groups by work-related psychosocial factors were mainly explained by the morning BP values and not by the evening BP values. There are several possible explanations for this finding. As all the participants had a regular daytime job, their morning BP could have been measured in a rush, leading to increased morning BP values. On the contrary, the evening BP values did not differ between weekdays and weekend days. This finding could have been a result of work-related psychosocial factors having a smaller impact on evening BP values as BP was measured several hours after the workday. Moreover, participants with sleep apnea have been previously shown to have higher morning BP values compared to the evening BP values [19] and participants with insomnia or long sleep duration (9 h or more) have been found to have higher morning-evening home BP variability [20]. Therefore, sleep apnea and other sleep disorders could perhaps account for the differences between morning and evening BP values in this study, too.

Interestingly, we found that high job strain and high job demands, but not low job control, were related to a reduction in BP from weekdays to the weekend. Assuming that the majority of the participants worked on weekdays and had weekends off, we can speculate that this difference depends on the characteristics of the different occupations. As high job demands seem to associate with higher BP levels during weekdays than during weekend days, we may consider that the participants in active jobs (with high job demands and high job control, such as public officials, physicians, engineers, nurses and managers) and the participants in high strain jobs (assemblers, cutting operatives, freight handlers and waiters) are at the highest risk for work-related BP increases. However, participants in passive jobs (with low job demands and low job control; billing clerks, janitors) and the participants in low strain jobs (with low job demands and high job control; repairmen, linemen, natural scientists) have the lowest work-related risk for BP increase. In this case, it can be assumed that work-related psychosocial factors have a short-term effect on BP as we observe BP variation within a week's time period.

The main strengths of the current study include a representative, population-based sample with a wide variety of professions and seven days home BP measurements. In addition, the results of this study are generalizable to the larger population as the current study sample did not differentiate from the overall participants of the nationally representative Health 2000 Study regarding BPs, life-style factors or work-related psychosocial factors. Moreover, the results remained significant after adjusting for a number of relevant covariates. Based on these covariates, it is also possible to state that the potentially different lifestyles between weekdays and weekend, such as physical activity and alcohol use, did not affect our results. In addition, our study sample was relatively large (*N* = 754) and included both sexes. Furthermore, as the mean age of the participants was 51 years, we can assume that the majority of participants were experienced workers and had probably held the same job for a long time. Additionally, home-measured BP provides an inexpensive and widely available method for multiple BP measurements during several days and is helpful in diagnosing white-coat and masked hypertension [11,21]. Moreover, the results indicated that the differences in BP changes attributed mostly to the morning BP values and not to the evening BP values. Finally, we also examined the role of working-time mode and observed, that the full-time workers did not differ from the part-time workers regarding home-measured BPs during a week.

A limitation of this study is that we cannot be certain if all participants were working from Monday to Friday and had days off on Saturday and Sunday, as the requirement for the regular job does not guarantee that the working days were weekdays and not weekend days, although this is usually the case. Moreover, we did not have information about the daily activities or working hours of the participants, which hampers the interpretation of the between group differences in BPs. In addition, as home BP measurements were used to assess BP, we could not determine, whether the work-related psychosocial factors are related to nocturnal BP and nocturnal BP dipping that are particularly strong predictors of cardiovascular events [22]. Furthermore, the participants were not blinded to the home BP measurements. Moreover, from the clinical perspective, the differences in BP between weekdays and weekend remained mainly negligible, as a 10/5 mmHg elevation of systolic home BP relates to a 29%/18% greater risk of stroke [23].

## CONCLUSION

High job strain and high job demands were associated with a greater BP reduction from weekdays to the weekend. Work-related factors should be considered when assessing day-to-day BP variation and BP level.

## ACKNOWLEDGEMENTS

The Health 2000 Study participants are thanked for the attendance at the study and the Health 2000 staff members are thanked for the contribution to the data collection.

S.K., T.J.N. and S.S. conceived and designed the analysis. A.J., S.Ko., T.J.N. collected the data. S.K., E.J. and J.P. performed the analysis. S.K., T.J.N., and S.S. wrote the article. E.J., A.J., J.P. and S.Ko. revised the manuscript critically for important intellectual content.

The phenotype data contain sensitive information from healthcare registers and they are available through the THL biobank upon submission of a research plan and signing a data transfer agreement (https://thl.fi/en/web/thl-biobank/for-researchers/application-process).

This work was financially supported by the State Research Funding (to S.K., S.S.), the Turku University Foundation (to S.K.), Academy of Finland (332030 to S.S. and 321351 to T.J.N.), the Sigrid Jusélius Foundation (to T.J.N.) and the Finnish Foundation for Cardiovascular Research (to T.J.N.).

### Conflicts of interest

The authors state no conflicts of interest.

## Supplementary Material

Supplemental Digital Content
