# Supplementary material for: Association of work-related psychosocial factors and day-to-day home blood pressure variation: the Finn-Home study
Source: J Hypertens. 2023 Nov 15;42(2):337–43. doi: 10.1097/HJH.0000000000003619 (PMC10763707; doi:10.1097/HJH.0000000000003619)
Supplement: Supplemental Digital Content [file jhype-42-337-s001.doc]

Supplementary Table 1. Characteristics of the study participants by work-related psychosocial factor.

| **Characteristic** | **Job strain** | | |  | **Job demands** | | |  | **Job control** | | |
| --- | --- | --- | --- | --- | --- | --- | --- | --- | --- | --- | --- |
|  | Low | High | P-value |  | Low | High | P-value |  | Low | High | P-value |
| **Women (n, %)** | 284 (48) | 97 (58) | 0.027 |  | 207 (49) | 174 (53) | 0.25 |  | 216 (58) | 165 (43) | <0.0001 |
| **Age (mean, SD)** | 50.9 (4.8) | 50.9 (4.9) | 0.97 |  | 51.0 (4.9) | 50.8 (4.8) | 0.46 |  | 50.9 (4.9) | 51.0 (4.7) | 0.85 |
| **Low education (n, %)** | 134 (23) | 68 (41) | <0.0001 |  | 105 (25) | 97 (29) | 0.071 |  | 132 (35) | 70 (18) | <0.0001 |
| **Manual occupation (n, %)** | 102 (17) | 44 (26) | <0.0001 |  | 83 (20) | 63 (19) | 0.84 |  | 92 (25) | 54 (14) | <0.0001 |
| **BMI (kg/m^2^) (mean, SD)** | 27.1 (4.3) | 26.5 (4.0) | 0.11 |  | 27.0 (4.4) | 26.8 (4.1) | 0.57 |  | 26.6 (4.2) | 27.3 (4.3) | 0.019 |
| **Low physical activity (n, %)** | 144 (25) | 36 (22) | 0.21 |  | 107 (25) | 73 (22) | 0.39 |  | 95 (25) | 85 (22) | 0.11 |
| **Excessive alcohol consumption (n, %)** | 57 (10) | 7 (4) | 0.023 |  | 40 (9) | 24 (7) | 0.29 |  | 28 (7) | 36 (10) | 0.32 |
| **Current smoker (n, %)** | 147 (25) | 46 (28) | 0.51 |  | 106 (25) | 87 (26) | 0.64 |  | 98 (26) | 95 (25) | 0.71 |
| **Antihypertensive medication (n, %)** | 104 (20) | 38 (23) | 0.31 |  | 76 (20) | 66 (21) | 0.66 |  | 67 (19) | 75 (22) | 0.44 |
| **Hypertension (n, %)** | 187 (32) | 58 (35) | 0.48 |  | 134 (32) | 111 (34) | 0.52 |  | 120 (32) | 125 (33) | 0.81 |

Data is presented as number of participants (percentage) for class variables or as mean (SD) for continuous variables.

SD Standard deviation BMI Body mass index

**Supplementary Table 2.** **Comparison between the study participants and the health examination participants.**

| **Characteristic** | **Study Participants (n=754)** | **Health examination participants (n=1212)** |
| --- | --- | --- |
| **Women (n, %)** | 381 (51) | 620 (51) |
| **Age (mean, SD)** | 50.9±4.8 | 51.6±4.6 |
| **Low education (n, %)**  **Manual occupation (n, %)** | 202 (27)  146 (19) | 347 (29)  254 (21) |
| **High job demands (n, %)** | 329 (44) | 543 (45) |
| **Low job control (n, %)** | 374 (50) | 616 (51) |
| **High job strain (n, %)** | 167 (22) | 286 (24) |
| **BMI (kg/m^2^) (mean, SD)** | 26.9±4.3 | 27.0±4.3 |
| **Low physical activity (n, %)** | 180 (24) | 290 (24) |
| **Excessive alcohol consumption (n, %)** | 64 (9) | 109 (9) |
| **Current smoker (n, %)** | 193 (26) | 305 (25) |
| **Antihypertensive medication (n, %)** | 142 (21) | 216 (20) |
| **Office systolic BP (mean, SD)** | 132.3±17.7 | 133.7± 18.7 |
| **Office diastolic BP (mean, SD)** | 83.8±10.7 | 84.0± 10.5 |
| **Hypertension (n, %)** | 245 (32) | 363 (30) |

Data is presented as number of participants (percentage) for class variables or as mean (SD) for continuous variables.

*SD* Standard deviation *BMI* Body mass index *BP* Blood pressure

Supplementary Table 3. Mean level and change between weekdays and weekend BP by work-related psychosocial factor.

| **BP** | **Work-related psychosocial factor** | **Weekday BP**  **mean (95% CI)** | **Weekend BP**  **mean (95% CI)** | **BP change between weekdays and weekend**  **mean (95% CI)** | **P for interaction time*work-related psychosocial factor** |
| --- | --- | --- | --- | --- | --- |
|  | **Job strain** | | | | |
| **Systolic** | Low | 125.3 (123.5 to 127.0) | 124.6 (122.8 to 126.4) | -0.7 (-1.1 to -0.2) | 0.038 |
|  | High | 125.5 (122.8 to 128.2) | 123.8 (121.1 to 126.4) | -1.8 (-2.7 to -0.8) |  |
| **Diastolic** | Low | 80.0 (79.0 to 81.0) | 79.3 (78.3 to 80.3) | -0.7 (-1.0 to -0.4) | 0.0027 |
|  | High | 79.3 (77.9 to 80.8) | 77.6 (76.2 to 79.1) | -1.7 (-2.3 to -1.1) |  |
|  | **Job demands** | | | | |
| **Systolic** | Low | 125.4 (123.5 to 127.2) | 124.8 (123.0 to 126.7) | -0.5 (-1.1 to 0.0) | 0.034 |
|  | High | 125.2 (123.1 to 127.4) | 123.8 (121.6 to 126.0) | -1.4 (-2.0 to -0.8) |  |
| **Diastolic** | Low | 79.9 (78.9 to 80.9) | 79.3 (78.2 to 80.3) | -0.6 (-1.0 to -0.3) | 0.019 |
|  | High | 79.5 (78.3 to 80.7) | 78.3 (77.1 to 79.4) | -1.3 (-1.6 to -0.9) |  |
|  | **Job control** | | | | |
| **Systolic** | Low | 125.5 (123.7 to 127.4) | 124.6 (122.7 to 126.5) | -0.9 (-1.5 to -0.3) | 0.91 |
|  | High | 124.9 (122.8 to 127.0) | 124.0 (121.9 to 126.2) | -0.9 (-1.5 to -0.3) |  |
| **Diastolic** | Low | 79.7 (78.7 to 80.8) | 78.8 (77.8 to 79.9) | -0.9 (-1.3 to -0.5) | 0.92 |
|  | High | 79.7 (78.5 to 80.9) | 78.8 (77.6 to 80.0) | -0.9 (-1.3 to -0.6) |  |

Means are adjusted for age, sex, educational level and occupational status.

*BP* Blood pressure *CI* Confidence interval

Supplementary Table 4. Mean level and change between weekdays and weekend BP by work-related psychosocial factor among participants with full-time employment (n=707).

| **BP** | **Work-related psychosocial factor** | **Weekday BP**  **mean (95% CI)** | **Weekend BP**  **mean (95% CI)** | **BP change between weekdays and weekend**  **mean (95% CI)** | **P for interaction time*work-related psychosocial factor** |
| --- | --- | --- | --- | --- | --- |
|  | **Job strain** | | | | |
| **Systolic** | Low | 127.7 (125.4 to 130.0) | 127.0 (124.7 to 129.3) | -0.6 (-1.1 to -0.2) | 0.033 |
|  | High | 129.1 (125.8 to 132.4) | 127.3 (124.1 to 130.4) | -1.8 (-2.8 to -0.9) |  |
| **Diastolic** | Low | 81.7 (80.4 to 83.0) | 81.0 (79.7 to 82.3) | -0.7 (-1.0 to -0.4) | 0.0010 |
|  | High | 81.7 (79.9 to 83.4) | 79.8 (78.1 to 81.5) | -1.9 (-2.5 to -1.3) |  |
|  | **Job demands** | | | | |
| **Systolic** | Low | 128.1 (125.7 to 130.4) | 127.6 (125.2 to 129.9) | -0.5 (-1.1 to 0.09) | 0.028 |
|  | High | 127.9 (125.2 to 130.6) | 126.5 (123.8 to 129.2) | -1.4 (-2.1 to -0.8) |  |
| **Diastolic** | Low | 81.9 (80.5 to 83.2) | 81.2 (79.8 to 82.6) | -0.7 (-1.0 to -0.3) | 0.015 |
|  | High | 81.4 (79.9 to 82.9) | 80.1 (78.6 to 81.6) | -1.3 (-1.7 to -0.9) |  |
|  | **Job control** | | | | |
| **Systolic** | Low | 128.8 (126.2 to 131.3) | 127.8 (125.2 to 130.3) | -1.0 (-1.6 to -0.4) | 0.76 |
|  | High | 126.9 (124.4 to 129.5) | 126.1 (123.6 to 128.6) | -0.8 (-1.4 to -0.3) |  |
| **Diastolic** | Low | 82.0 (80.5 to 83.4) | 81.0 (79.6 to 82.5) | -1.0 (-1.4 to -0.6) | 0.98 |
|  | High | 81.2 (79.8 to 82.7) | 80.3 (78.8 to 81.7) | -1.0 (-1.3 to -0.6) |  |

Means are adjusted for age, sex, educational level, occupational status, BMI, physical activity, smoking and alcohol use.

*BP* Blood pressure *CI* Confidence interval
